# Dimethyl 1,8-bis­(4-methyl­phen­yl)-11-oxatri­cyclo­[6.2.1.0^2,7^]undeca-2,4,6,9-tetra­ene-9,10-di­carboxyl­ate

**DOI:** 10.1107/S1600536813013305

**Published:** 2013-05-25

**Authors:** B. Balakrishnan, Meganathan Nandakumar, P. R. Seshadri, Arasambattu K. Mohanakrishnan

**Affiliations:** a Department of Physics, P.T. Lee Chengalvaraya Naicker College of Engineering and Technology, Kancheepuram 631 502, India; b Department of Organic Chemistry, University of Madras, Guindy Campus, Chennai 600 025, India; c Postgraduate and Research Department of Physics, Agurchand Manmull Jain College, Chennai 600 114, India

## Abstract

The title compound, C_28_H_24_O_5_, consists of a fused tricyclic system containing two five-membered rings and one six-membered ring. The five-membered rings both exhibit an envelope conformation with the O atom at the flap, whereas the six-membered ring adopts a boat conformation. The dihedral angle between the 4-methyl­phenyl rings at the 1,8-positions is 76.4 (1)°. In the crystal, mol­ecules are stacked in columns along the *a* axis through C—H⋯O inter­actions.

## Related literature

For background to Diels–Alder reactions, see: Denmark & Thorarensen (1996 ▶). For related structures, see: Bailey *et al.* (1995 ▶); Balakrishnan *et al.* (2013 ▶); Takahashi *et al.* (2003 ▶). For puckering and asymmetry parameters, see: Cremer & Pople (1975 ▶); Nardelli (1983 ▶).
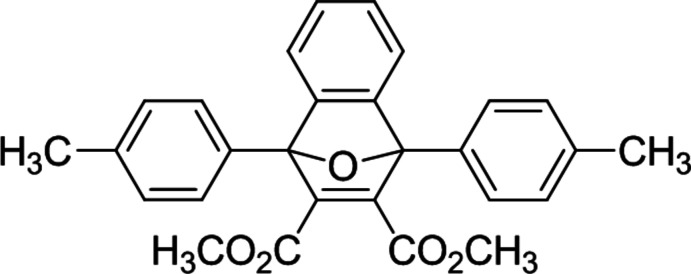


## Experimental

### 

#### Crystal data

C_28_H_24_O_5_
*M* *_r_* = 440.47
Monoclinic, 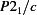

*a* = 8.9018 (4) Å
*b* = 25.6357 (8) Å
*c* = 11.0961 (4) Å
β = 113.369 (1)°
*V* = 2324.46 (15) Å^3^
*Z* = 4
Mo *K*α radiation
μ = 0.09 mm^−1^
*T* = 293 K
0.30 × 0.20 × 0.20 mm

#### Data collection

Bruker Kappa APEXII CCD diffractometer
Absorption correction: multi-scan (*SADABS*; Bruker, 2004 ▶) *T*_min_ = 0.975, *T*_max_ = 0.983
29434 measured reflections
6904 independent reflections
4338 reflections with *I* > 2σ(*I*)
*R*_int_ = 0.034

#### Refinement

*R*[*F* ^2^ > 2σ(*F* ^2^)] = 0.055
*wR*(*F* ^2^) = 0.170
*S* = 1.03
6904 reflections
299 parameters
H-atom parameters constrained
Δρ_max_ = 0.30 e Å^−3^
Δρ_min_ = −0.17 e Å^−3^

### 

Data collection: *APEX2* (Bruker, 2004 ▶); cell refinement: *SAINT* (Bruker, 2004 ▶); data reduction: *SAINT*; program(s) used to solve structure: *SHELXS97* (Sheldrick, 2008 ▶); program(s) used to refine structure: *SHELXL97* (Sheldrick, 2008 ▶); molecular graphics: *ORTEP-3 for Windows* (Farrugia, 2012 ▶) and *PLATON* (Spek, 2009 ▶); software used to prepare material for publication: *SHELXL97*, *PLATON* and *publCIF* (Westrip, 2010 ▶).

## Supplementary Material

Crystal structure: contains datablock(s) I, global. DOI: 10.1107/S1600536813013305/is5270sup1.cif

Structure factors: contains datablock(s) I. DOI: 10.1107/S1600536813013305/is5270Isup2.hkl

Supplementary material file. DOI: 10.1107/S1600536813013305/is5270Isup3.cml

Additional supplementary materials:  crystallographic information; 3D view; checkCIF report

## Figures and Tables

**Table 1:** Hydrogen-bond geometry (Å, °)

*D*—H⋯*A*	*D*—H	H⋯*A*	*D*⋯*A*	*D*—H⋯*A*
C13—H13*C*⋯O3^i^	0.96	2.56	3.461 (3)	157
C28—H28*B*⋯O3^ii^	0.96	2.52	3.478 (3)	173

Symmetry codes: (i) 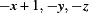; (ii) 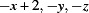.

**Fig. 1.**
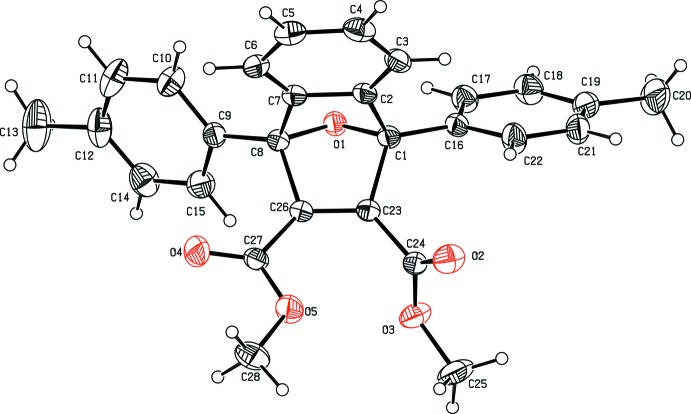
Molecular structure of the title compound, showing 30% probability displacement ellipsoids.

**Fig. 2.**
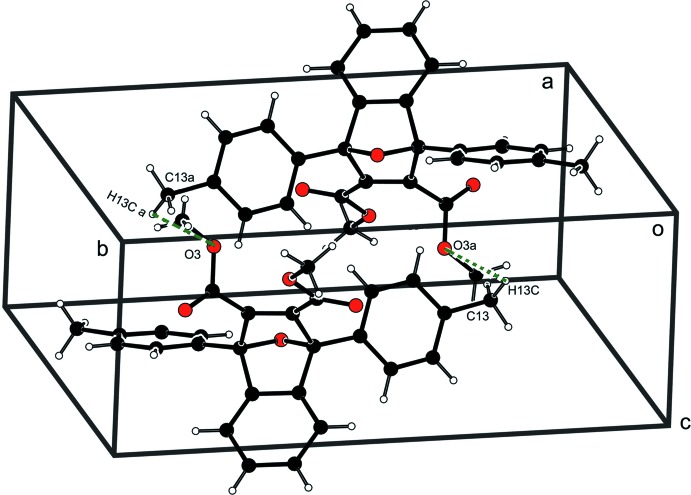
Crystal packing of the title compound. Hydrogen bonds are shown as dashed lines.

## supplementary crystallographic information

### Comment

The Diels-Alder reaction is among the most powerful C—C bond forming process and one of the most widely used and studied transformation in organic chemistry. The isobenzofuran have found extensive use as reactive diene in intra and inter molecular Diels-Alder reactions for the rapid construction of poly cyclic ring (Denmark & Thorarensen, 1996).

The title compound, C_28_H_24_O_5_, comprises a fused tricyclic system and two 4-methylphenyl rings attached to this system (Fig. 1). The tricyclic system consists of two 5-membered rings and one aromatic ring. In addition, two carboxylate units are attached to the tricyclic system. Geometrical parameters agree well with reported structures (Bailey *et al.*, 1995; Takahashi *et al.*, 2003; Balakrishnan *et al.*, 2013). The 5-membered ring C1/C2/C7/C8/O1 adopts an envelope conformation with atom O1 displaced by -0.772 Å from the mean plane of the other ring atoms C1/C2/C7/C8. The puckering parameters (Cremer & Pople, 1975) and asymmetry parameters (Nardelli, 1983) are q_2_ = 0.530 (1) Å, φ = 143.5 (2)°, Δ_S_(O1) = 0.004 (1)° and Δ_2_(O1) = 0.319 (1)°. The second 5-membered ring C1/C23/C26/C8/O1 also adopts an envelope conformation with atom O1 displaced by -0.776 Å from the mean plane of the other ring atoms C1/C23/C26/C8. The puckering parameters (Cremer & Pople, 1975) and asymmetry parameters (Nardelli, 1983) are q_2_ = 0.556 (1) Å, φ = -33.2 (2)°, Δ_S_(O1) = 0.022 (1)° and Δ_2_(O1) = 0.323 (1)°. The six membered ring C1/C2/C7/C8/C26/C23 adopts a boat conformation with puckering parameter q_2_ = 0.999 (1) Å, θ = 90.4 (1)° and φ = 1.1 (1)°.

The dihedral angle between the mean planes of the rings C1/C2/C7/C8/O1 and C1/C23/C26/C8/O1 is 72.5 (1)°. The mean plane of the tricyclic system makes dihedral angles of 31.4 (1) and 69.9 (1)°, respectively, with the 4-methylphenyl rings. The dihedral angle between the terminal 4-methylphenyl rings is 76.4 (1). The carboxylate ligand at the C26 carbon atom lies practically in the plane of the C26/C8/O1/C1 five ring [C23—C26—C27—O5 torsion angle = -7.1 (2)°], while that at the C23 carbon atom is turned out of this plane [C26—C23—C24—O3 torsion angle = 72.0 (2)°]. Centrosymmetric dimers are formed by C—H···O hydrogen bond.

### Experimental

Diels-Alder reaction of 1,3-di-*p*-tolylbenzo[*c*]furan (1.0 g, 3.36 mmol) in dry DCM was added DMAD (0.53 g, 3.69 mmol) and the reaction mixture was stirred for 0.5 h at room temperature under nitrogen atmosphere. The solvent was removed and solid was washed with methanol to give compound as colourless solid. Yield: 1.07 g (80%). M.P.: 116 °C. This adduct was crystallized from CHCl_3_/CH_3_OH (3:1) by the slow evaporation method.

### Refinement

All H atoms were positioned geometrically and allowed to ride on their parent atoms, with (C—H = 0.93–0.96 Å), and with *U*_iso_(H) = 1.5*U*_eq_(C) for methyl H atoms and 1.2*U*_eq_(C) for other H atoms.

### Crystal data

**Table:**

C_28_H_24_O_5_	*Z* = 4
*M**_r_* = 440.47	*F*(000) = 928
Monoclinic, *P*2_1_/*c*	*D*_x_ = 1.259 Mg m^−^^3^
Hall symbol: -P 2ybc	Mo *K*α radiation, λ = 0.71073 Å
*a* = 8.9018 (4) Å	θ = 2.2–30.2°
*b* = 25.6357 (8) Å	µ = 0.09 mm^−^^1^
*c* = 11.0961 (4) Å	*T* = 293 K
β = 113.369 (1)°	Black, colourless
*V* = 2324.46 (15) Å^3^	0.30 × 0.20 × 0.20 mm

### Data collection

**Table:**

Bruker Kappa APEXII CCD diffractometer	6904 independent reflections
Radiation source: fine-focus sealed tube	4338 reflections with *I* > 2σ(*I*)
Graphite monochromator	*R*_int_ = 0.034
ω and φ scan	θ_max_ = 30.2°, θ_min_ = 2.2°
Absorption correction: multi-scan (*SADABS*; Bruker, 2004)	*h* = −12→12
*T*_min_ = 0.975, *T*_max_ = 0.983	*k* = −36→34
29434 measured reflections	*l* = −15→15

### Refinement

**Table:**

Refinement on *F*^2^	Secondary atom site location: difference Fourier map
Least-squares matrix: full	Hydrogen site location: inferred from neighbouring sites
*R*[*F*^2^ > 2σ(*F*^2^)] = 0.055	H-atom parameters constrained
*wR*(*F*^2^) = 0.170	*w* = 1/[σ^2^(*F*_o_^2^) + (0.0787*P*)^2^ + 0.4566*P*] where *P* = (*F*_o_^2^ + 2*F*_c_^2^)/3
*S* = 1.03	(Δ/σ)_max_ < 0.001
6904 reflections	Δρ_max_ = 0.30 e Å^−^^3^
299 parameters	Δρ_min_ = −0.17 e Å^−^^3^
0 restraints	Extinction correction: *SHELXL97* (Sheldrick, 2008), Fc^*^=kFc[1+0.001xFc^2^λ^3^/sin(2θ)]^-1/4^
Primary atom site location: structure-invariant direct methods	Extinction coefficient: 0.0071 (14)

### Special details

**Table:**

Geometry. All e.s.d.'s (except the e.s.d. in the dihedral angle between two l.s. planes) are estimated using the full covariance matrix. The cell e.s.d.'s are taken into account individually in the estimation of e.s.d.'s in distances, angles and torsion angles; correlations between e.s.d.'s in cell parameters are only used when they are defined by crystal symmetry. An approximate (isotropic) treatment of cell e.s.d.'s is used for estimating e.s.d.'s involving l.s. planes.
Refinement. Refinement of *F*^2^ against ALL reflections. The weighted *R*-factor *wR* and goodness of fit *S* are based on *F*^2^, conventional *R*-factors *R* are based on *F*, with *F* set to zero for negative *F*^2^. The threshold expression of *F*^2^ > σ(*F*^2^) is used only for calculating *R*-factors(gt) *etc*. and is not relevant to the choice of reflections for refinement. *R*-factors based on *F*^2^ are statistically about twice as large as those based on *F*, and *R*- factors based on ALL data will be even larger.

### Fractional atomic coordinates and isotropic or equivalent isotropic displacement parameters (Å^2^)

**Table:**

	*x*	*y*	*z*	*U*_iso_*/*U*_eq_	
C8	0.77122 (19)	0.06795 (6)	0.26149 (15)	0.0371 (3)	
C7	0.8610 (2)	0.10788 (6)	0.36867 (16)	0.0398 (3)	
C6	0.9676 (2)	0.10415 (7)	0.49831 (16)	0.0465 (4)	
H6	0.9919	0.0721	0.5411	0.056*	
C5	1.0378 (2)	0.15018 (8)	0.56286 (18)	0.0537 (5)	
H5	1.1099	0.1488	0.6506	0.064*	
C4	1.0032 (2)	0.19756 (8)	0.5002 (2)	0.0549 (5)	
H4	1.0505	0.2277	0.5465	0.066*	
C3	0.8982 (2)	0.20119 (7)	0.36820 (18)	0.0479 (4)	
H3	0.8759	0.2332	0.3252	0.057*	
C2	0.82902 (19)	0.15609 (6)	0.30388 (16)	0.0391 (3)	
C1	0.71914 (19)	0.14347 (6)	0.15944 (15)	0.0380 (3)	
C16	0.60917 (19)	0.18477 (6)	0.07505 (16)	0.0409 (4)	
C22	0.6743 (2)	0.22872 (7)	0.0416 (2)	0.0570 (5)	
H22	0.7873	0.2329	0.0737	0.068*	
C21	0.5732 (3)	0.26660 (8)	−0.0392 (2)	0.0621 (5)	
H21	0.6193	0.2957	−0.0613	0.075*	
C19	0.4047 (2)	0.26197 (8)	−0.08750 (18)	0.0559 (5)	
C20	0.2962 (3)	0.30280 (11)	−0.1783 (2)	0.0864 (8)	
H20A	0.1836	0.2934	−0.2025	0.130*	
H20C	0.3191	0.3051	−0.2557	0.130*	
H20B	0.3169	0.3360	−0.1345	0.130*	
C18	0.3410 (2)	0.21909 (8)	−0.0500 (2)	0.0580 (5)	
H18	0.2281	0.2158	−0.0792	0.070*	
C17	0.4401 (2)	0.18069 (7)	0.03012 (18)	0.0499 (4)	
H17	0.3935	0.1521	0.0539	0.060*	
C9	0.7112 (2)	0.01647 (6)	0.28848 (16)	0.0420 (4)	
C10	0.6997 (2)	0.00459 (8)	0.40519 (19)	0.0574 (5)	
H10	0.7347	0.0286	0.4735	0.069*	
C11	0.6362 (3)	−0.04307 (10)	0.4220 (2)	0.0726 (6)	
H11	0.6310	−0.0506	0.5022	0.087*	
C12	0.5807 (2)	−0.07938 (8)	0.3224 (2)	0.0665 (6)	
C13	0.5158 (3)	−0.13196 (10)	0.3413 (3)	0.1046 (11)	
H13A	0.5200	−0.1343	0.4289	0.157*	
H13B	0.5816	−0.1591	0.3276	0.157*	
H13C	0.4046	−0.1358	0.2794	0.157*	
C14	0.5881 (3)	−0.06670 (8)	0.2050 (2)	0.0650 (5)	
H14	0.5488	−0.0902	0.1356	0.078*	
C15	0.6529 (2)	−0.01967 (7)	0.1876 (2)	0.0554 (5)	
H15	0.6574	−0.0122	0.1071	0.066*	
C26	0.87077 (18)	0.06868 (6)	0.17415 (14)	0.0363 (3)	
C27	1.0114 (2)	0.03366 (6)	0.19642 (16)	0.0407 (4)	
C28	1.2486 (2)	0.02100 (9)	0.1527 (2)	0.0694 (6)	
H28A	1.2993	0.0355	0.0984	0.104*	
H28B	1.2249	−0.0152	0.1315	0.104*	
H28C	1.3214	0.0243	0.2434	0.104*	
C23	0.83616 (18)	0.11356 (6)	0.10885 (14)	0.0359 (3)	
C24	0.91045 (19)	0.13555 (6)	0.02149 (15)	0.0380 (3)	
C25	0.9345 (4)	0.12516 (10)	−0.1798 (2)	0.0781 (7)	
H25A	0.8917	0.1039	−0.2573	0.117*	
H25B	1.0511	0.1209	−0.1384	0.117*	
H25C	0.9091	0.1611	−0.2033	0.117*	
O1	0.63095 (13)	0.09913 (4)	0.17856 (11)	0.0401 (3)	
O4	1.0475 (2)	−0.00215 (6)	0.27114 (16)	0.0750 (5)	
O5	1.09867 (17)	0.04857 (6)	0.13005 (15)	0.0648 (4)	
O2	0.99992 (17)	0.17240 (5)	0.04755 (13)	0.0595 (4)	
O3	0.86178 (17)	0.10946 (5)	−0.08975 (12)	0.0566 (4)	

### Atomic displacement parameters (Å^2^)

**Table:**

	*U*^11^	*U*^22^	*U*^33^	*U*^12^	*U*^13^	*U*^23^
C8	0.0394 (8)	0.0369 (8)	0.0392 (8)	0.0058 (6)	0.0199 (7)	0.0033 (6)
C7	0.0438 (8)	0.0403 (8)	0.0424 (8)	0.0035 (7)	0.0246 (7)	−0.0017 (6)
C6	0.0512 (10)	0.0517 (10)	0.0426 (9)	0.0039 (8)	0.0250 (8)	0.0009 (7)
C5	0.0534 (10)	0.0666 (12)	0.0443 (9)	−0.0025 (9)	0.0227 (8)	−0.0100 (9)
C4	0.0575 (11)	0.0533 (11)	0.0607 (11)	−0.0079 (9)	0.0307 (9)	−0.0196 (9)
C3	0.0523 (10)	0.0391 (8)	0.0613 (11)	0.0028 (7)	0.0321 (9)	−0.0042 (8)
C2	0.0403 (8)	0.0382 (8)	0.0464 (8)	0.0055 (6)	0.0252 (7)	−0.0011 (6)
C1	0.0376 (8)	0.0360 (8)	0.0465 (8)	0.0027 (6)	0.0230 (7)	0.0026 (6)
C16	0.0398 (8)	0.0394 (8)	0.0484 (9)	0.0075 (6)	0.0228 (7)	0.0041 (7)
C22	0.0434 (9)	0.0532 (11)	0.0804 (13)	0.0116 (8)	0.0308 (9)	0.0201 (10)
C21	0.0627 (12)	0.0557 (11)	0.0822 (14)	0.0194 (9)	0.0438 (11)	0.0275 (10)
C19	0.0558 (11)	0.0655 (12)	0.0493 (10)	0.0225 (9)	0.0239 (9)	0.0141 (9)
C20	0.0793 (16)	0.1036 (19)	0.0741 (15)	0.0402 (15)	0.0280 (13)	0.0407 (14)
C18	0.0397 (9)	0.0655 (12)	0.0632 (12)	0.0108 (9)	0.0146 (9)	0.0046 (10)
C17	0.0450 (9)	0.0467 (9)	0.0602 (11)	0.0031 (7)	0.0230 (8)	0.0026 (8)
C9	0.0424 (8)	0.0381 (8)	0.0486 (9)	0.0040 (7)	0.0214 (7)	0.0058 (7)
C10	0.0621 (12)	0.0612 (11)	0.0511 (10)	−0.0081 (9)	0.0248 (9)	0.0072 (9)
C11	0.0665 (13)	0.0842 (16)	0.0634 (13)	−0.0132 (12)	0.0219 (11)	0.0292 (12)
C12	0.0456 (10)	0.0532 (11)	0.0907 (16)	−0.0019 (9)	0.0164 (10)	0.0258 (11)
C13	0.0777 (17)	0.0762 (16)	0.138 (3)	−0.0227 (14)	0.0192 (17)	0.0424 (17)
C14	0.0620 (12)	0.0479 (10)	0.0832 (15)	−0.0097 (9)	0.0268 (11)	−0.0047 (10)
C15	0.0648 (12)	0.0475 (10)	0.0594 (11)	−0.0070 (9)	0.0306 (10)	−0.0042 (8)
C26	0.0387 (8)	0.0355 (7)	0.0377 (7)	0.0030 (6)	0.0183 (6)	−0.0014 (6)
C27	0.0432 (9)	0.0404 (8)	0.0387 (8)	0.0068 (7)	0.0165 (7)	−0.0037 (7)
C28	0.0493 (11)	0.0777 (14)	0.0899 (16)	0.0173 (10)	0.0368 (11)	−0.0038 (12)
C23	0.0345 (7)	0.0376 (8)	0.0376 (8)	0.0012 (6)	0.0163 (6)	−0.0017 (6)
C24	0.0364 (8)	0.0394 (8)	0.0402 (8)	0.0020 (6)	0.0171 (6)	0.0003 (6)
C25	0.1121 (19)	0.0845 (15)	0.0655 (13)	−0.0214 (14)	0.0648 (14)	−0.0108 (12)
O1	0.0375 (6)	0.0379 (6)	0.0494 (6)	0.0030 (4)	0.0218 (5)	0.0054 (5)
O4	0.0805 (10)	0.0741 (9)	0.0850 (10)	0.0389 (8)	0.0484 (9)	0.0360 (8)
O5	0.0582 (8)	0.0647 (8)	0.0903 (10)	0.0230 (7)	0.0495 (8)	0.0212 (7)
O2	0.0621 (8)	0.0579 (8)	0.0655 (8)	−0.0220 (7)	0.0329 (7)	−0.0116 (6)
O3	0.0732 (9)	0.0604 (8)	0.0466 (7)	−0.0216 (7)	0.0349 (6)	−0.0126 (6)

### Geometric parameters (Å, º)

**Table:**

C8—O1	1.4607 (18)	C17—H17	0.9300
C8—C9	1.498 (2)	C9—C10	1.373 (2)
C8—C7	1.532 (2)	C9—C15	1.386 (3)
C8—C26	1.551 (2)	C10—C11	1.390 (3)
C7—C6	1.377 (2)	C10—H10	0.9300
C7—C2	1.401 (2)	C11—C12	1.378 (3)
C6—C5	1.393 (3)	C11—H11	0.9300
C6—H6	0.9300	C12—C14	1.369 (3)
C5—C4	1.372 (3)	C12—C13	1.514 (3)
C5—H5	0.9300	C13—H13A	0.9600
C4—C3	1.393 (3)	C13—H13B	0.9600
C4—H4	0.9300	C13—H13C	0.9600
C3—C2	1.370 (2)	C14—C15	1.383 (3)
C3—H3	0.9300	C14—H14	0.9300
C2—C1	1.545 (2)	C15—H15	0.9300
C1—O1	1.4441 (18)	C26—C23	1.329 (2)
C1—C16	1.493 (2)	C26—C27	1.480 (2)
C1—C23	1.566 (2)	C27—O4	1.192 (2)
C16—C22	1.384 (2)	C27—O5	1.321 (2)
C16—C17	1.389 (2)	C28—O5	1.441 (2)
C22—C21	1.384 (3)	C28—H28A	0.9600
C22—H22	0.9300	C28—H28B	0.9600
C21—C19	1.383 (3)	C28—H28C	0.9600
C21—H21	0.9300	C23—C24	1.484 (2)
C19—C18	1.375 (3)	C24—O2	1.1948 (19)
C19—C20	1.507 (3)	C24—O3	1.3170 (19)
C20—H20A	0.9600	C25—O3	1.447 (2)
C20—H20C	0.9600	C25—H25A	0.9600
C20—H20B	0.9600	C25—H25B	0.9600
C18—C17	1.384 (3)	C25—H25C	0.9600
C18—H18	0.9300		
			
O1—C8—C9	108.93 (12)	C16—C17—H17	120.0
O1—C8—C7	100.11 (11)	C10—C9—C15	118.01 (16)
C9—C8—C7	123.44 (13)	C10—C9—C8	123.36 (16)
O1—C8—C26	99.05 (11)	C15—C9—C8	118.46 (15)
C9—C8—C26	118.25 (12)	C9—C10—C11	120.5 (2)
C7—C8—C26	103.04 (12)	C9—C10—H10	119.7
C6—C7—C2	120.85 (15)	C11—C10—H10	119.7
C6—C7—C8	134.08 (15)	C12—C11—C10	121.4 (2)
C2—C7—C8	104.71 (13)	C12—C11—H11	119.3
C7—C6—C5	117.45 (17)	C10—C11—H11	119.3
C7—C6—H6	121.3	C14—C12—C11	117.75 (18)
C5—C6—H6	121.3	C14—C12—C13	120.6 (2)
C4—C5—C6	121.63 (18)	C11—C12—C13	121.7 (2)
C4—C5—H5	119.2	C12—C13—H13A	109.5
C6—C5—H5	119.2	C12—C13—H13B	109.5
C5—C4—C3	120.92 (17)	H13A—C13—H13B	109.5
C5—C4—H4	119.5	C12—C13—H13C	109.5
C3—C4—H4	119.5	H13A—C13—H13C	109.5
C2—C3—C4	117.82 (17)	H13B—C13—H13C	109.5
C2—C3—H3	121.1	C12—C14—C15	121.3 (2)
C4—C3—H3	121.1	C12—C14—H14	119.3
C3—C2—C7	121.29 (16)	C15—C14—H14	119.3
C3—C2—C1	133.32 (15)	C14—C15—C9	120.90 (19)
C7—C2—C1	105.29 (13)	C14—C15—H15	119.6
O1—C1—C16	113.01 (12)	C9—C15—H15	119.6
O1—C1—C2	99.77 (12)	C23—C26—C27	127.89 (14)
C16—C1—C2	119.34 (13)	C23—C26—C8	106.55 (12)
O1—C1—C23	98.34 (11)	C27—C26—C8	122.93 (13)
C16—C1—C23	119.10 (13)	O4—C27—O5	123.69 (15)
C2—C1—C23	103.71 (12)	O4—C27—C26	124.04 (16)
C22—C16—C17	118.35 (16)	O5—C27—C26	112.14 (14)
C22—C16—C1	120.35 (15)	O5—C28—H28A	109.5
C17—C16—C1	121.29 (15)	O5—C28—H28B	109.5
C16—C22—C21	120.69 (18)	H28A—C28—H28B	109.5
C16—C22—H22	119.7	O5—C28—H28C	109.5
C21—C22—H22	119.7	H28A—C28—H28C	109.5
C19—C21—C22	121.19 (18)	H28B—C28—H28C	109.5
C19—C21—H21	119.4	C26—C23—C24	128.15 (14)
C22—C21—H21	119.4	C26—C23—C1	105.77 (12)
C18—C19—C21	117.71 (17)	C24—C23—C1	125.47 (13)
C18—C19—C20	121.7 (2)	O2—C24—O3	124.63 (15)
C21—C19—C20	120.6 (2)	O2—C24—C23	124.68 (14)
C19—C20—H20A	109.5	O3—C24—C23	110.67 (13)
C19—C20—H20C	109.5	O3—C25—H25A	109.5
H20A—C20—H20C	109.5	O3—C25—H25B	109.5
C19—C20—H20B	109.5	H25A—C25—H25B	109.5
H20A—C20—H20B	109.5	O3—C25—H25C	109.5
H20C—C20—H20B	109.5	H25A—C25—H25C	109.5
C19—C18—C17	121.92 (18)	H25B—C25—H25C	109.5
C19—C18—H18	119.0	C1—O1—C8	98.32 (11)
C17—C18—H18	119.0	C27—O5—C28	117.97 (15)
C18—C17—C16	120.06 (17)	C24—O3—C25	115.78 (15)
C18—C17—H17	120.0		
			
O1—C8—C7—C6	154.22 (17)	C15—C9—C10—C11	2.0 (3)
C9—C8—C7—C6	33.4 (3)	C8—C9—C10—C11	177.15 (18)
C26—C8—C7—C6	−103.93 (19)	C9—C10—C11—C12	−1.0 (3)
O1—C8—C7—C2	−32.90 (14)	C10—C11—C12—C14	−0.9 (3)
C9—C8—C7—C2	−153.74 (14)	C10—C11—C12—C13	178.2 (2)
C26—C8—C7—C2	68.95 (14)	C11—C12—C14—C15	1.7 (3)
C2—C7—C6—C5	2.0 (2)	C13—C12—C14—C15	−177.4 (2)
C8—C7—C6—C5	173.92 (16)	C12—C14—C15—C9	−0.6 (3)
C7—C6—C5—C4	−0.3 (3)	C10—C9—C15—C14	−1.3 (3)
C6—C5—C4—C3	−1.2 (3)	C8—C9—C15—C14	−176.61 (17)
C5—C4—C3—C2	1.0 (3)	O1—C8—C26—C23	30.30 (15)
C4—C3—C2—C7	0.7 (2)	C9—C8—C26—C23	147.58 (14)
C4—C3—C2—C1	−175.06 (16)	C7—C8—C26—C23	−72.38 (15)
C6—C7—C2—C3	−2.2 (2)	O1—C8—C26—C27	−166.84 (13)
C8—C7—C2—C3	−176.24 (14)	C9—C8—C26—C27	−49.6 (2)
C6—C7—C2—C1	174.58 (14)	C7—C8—C26—C27	90.48 (16)
C8—C7—C2—C1	0.54 (15)	C23—C26—C27—O4	168.97 (18)
C3—C2—C1—O1	−151.42 (17)	C8—C26—C27—O4	9.9 (3)
C7—C2—C1—O1	32.37 (14)	C23—C26—C27—O5	−7.1 (2)
C3—C2—C1—C16	−27.9 (2)	C8—C26—C27—O5	−166.12 (14)
C7—C2—C1—C16	155.86 (13)	C27—C26—C23—C24	12.2 (3)
C3—C2—C1—C23	107.43 (19)	C8—C26—C23—C24	173.96 (14)
C7—C2—C1—C23	−68.79 (14)	C27—C26—C23—C1	−159.09 (15)
O1—C1—C16—C22	−176.73 (15)	C8—C26—C23—C1	2.64 (16)
C2—C1—C16—C22	66.5 (2)	O1—C1—C23—C26	−35.12 (15)
C23—C1—C16—C22	−62.1 (2)	C16—C1—C23—C26	−157.38 (14)
O1—C1—C16—C17	4.4 (2)	C2—C1—C23—C26	67.14 (15)
C2—C1—C16—C17	−112.39 (18)	O1—C1—C23—C24	153.27 (14)
C23—C1—C16—C17	119.00 (17)	C16—C1—C23—C24	31.0 (2)
C17—C16—C22—C21	−2.6 (3)	C2—C1—C23—C24	−104.47 (16)
C1—C16—C22—C21	178.46 (18)	C26—C23—C24—O2	−109.2 (2)
C16—C22—C21—C19	0.7 (3)	C1—C23—C24—O2	60.5 (2)
C22—C21—C19—C18	1.6 (3)	C26—C23—C24—O3	72.0 (2)
C22—C21—C19—C20	−178.3 (2)	C1—C23—C24—O3	−118.32 (16)
C21—C19—C18—C17	−1.9 (3)	C16—C1—O1—C8	179.75 (12)
C20—C19—C18—C17	178.0 (2)	C2—C1—O1—C8	−52.41 (12)
C19—C18—C17—C16	0.0 (3)	C23—C1—O1—C8	53.15 (12)
C22—C16—C17—C18	2.3 (3)	C9—C8—O1—C1	−176.06 (12)
C1—C16—C17—C18	−178.81 (16)	C7—C8—O1—C1	53.18 (12)
O1—C8—C9—C10	−102.59 (18)	C26—C8—O1—C1	−51.92 (12)
C7—C8—C9—C10	14.1 (2)	O4—C27—O5—C28	−2.1 (3)
C26—C8—C9—C10	145.51 (17)	C26—C27—O5—C28	173.95 (16)
O1—C8—C9—C15	72.50 (18)	O2—C24—O3—C25	5.0 (3)
C7—C8—C9—C15	−170.83 (15)	C23—C24—O3—C25	−176.13 (17)
C26—C8—C9—C15	−39.4 (2)		

### Hydrogen-bond geometry (Å, º)

**Table:**

*D*—H···*A*	*D*—H	H···*A*	*D*···*A*	*D*—H···*A*
C13—H13*C*···O3^i^	0.96	2.56	3.461 (3)	157
C28—H28*B*···O3^ii^	0.96	2.52	3.478 (3)	173

Symmetry codes: (i) −*x*+1, −*y*, −*z*; (ii) −*x*+2, −*y*, −*z*.
